# Supplementary material for: Causal Relationship between Aging and Anorexia Nervosa: A White-Matter-Microstructure-Mediated Mendelian Randomization Analysis
Source: Biomedicines. 2024 Aug 16;12(8):1874. doi: 10.3390/biomedicines12081874 (PMC11351342; doi:10.3390/biomedicines12081874)
Supplement: Supplementary file 1 [file biomedicines-12-01874-s001.zip › Supplementary Figure/Figure S4/Singe SNP plot of MR analysis for TL to ubm-b-1753.pdf]

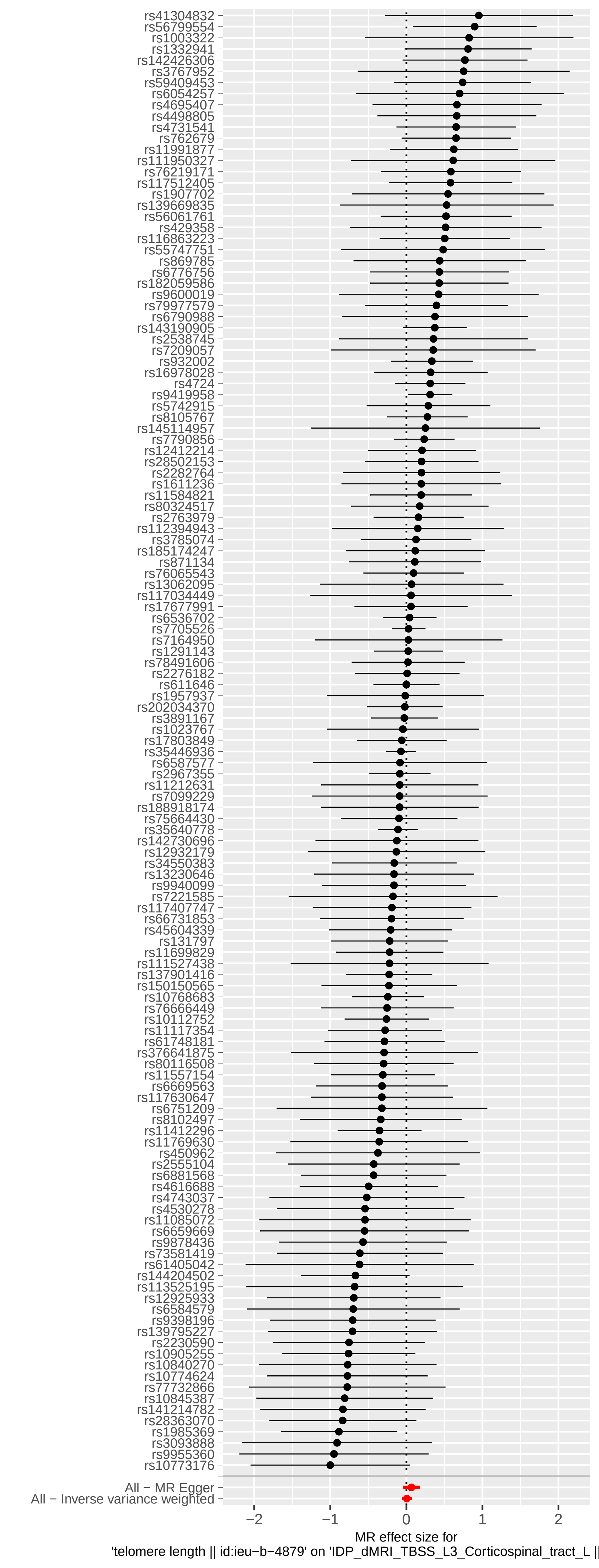
rs41304832
rs56799554
rs1003322
rs1332941
rs142426306
rs3767952
rs59409453
rs6054257
rs4695407
rs4498805
rs4731541
rs762679
rs11991877
rs111950327
rs76219171
rs117512405
rs1907702
rs139669835
rs56061761
rs429358
rs116863223
rs55747751
rs869785
rs6776756
rs182059586
rs9600019
rs79977579
rs6790988
rs143190905
rs2538745
rs7209057
rs932002
rs16978028
rs4724
rs9419958
rs5742915
rs8105767
rs145114957
rs7790856
rs12412214
rs28502153
rs2282764
rs1611236
rs11584821
rs80324517
rs2763979
rs112394943
rs3785074
rs185174247
rs871134
rs76065543
rs13062095
rs117034449
rs17677991
rs6536702
rs7705526
rs7164950
rs1291143
rs78491606
rs2276182
rs611646
rs1957937
rs202034370
rs3891167
rs1023767
rs17803849
rs35446936
rs6587577
rs2967355
rs11212631
rs7099229
rs188918174
rs75664430
rs35640778
rs142730696
rs12932179
rs34550383
rs13230646
rs9940099
rs7221585
rs117407747
rs66731853
rs45604339
rs131797
rs11699829
rs111527438
rs137901416
rs150150565
rs10768683
rs76666449
rs10112752
rs11117354
rs61748181
rs376641875
rs80116508
rs11557154
rs6669563
rs117630647
rs6751209
rs8102497
rs11412296
rs11769630
rs450962
rs2555104
rs6881568
rs4616688
rs4743037
rs4530278
rs11085072
rs6659669
rs9878436
rs73581419
rs61405042
rs144204502
rs113525195
rs12925933
rs6584579
rs9398196
rs139795227
rs2230590
rs10905255
rs10840270
rs10774624
rs77732866
rs10845387
rs141214782
rs28363070
rs1985369
rs3093888
rs9955360
rs10773176
All − MR Egger
All − Inverse variance weighted
−2
−1
0
1
2
MR effect size for
'telomere length || id:ieu−b−4879' on 'IDP_dMRI_TBSS_L3_Corticospinal_tract_L ||
